# Human-like conversational agents as social partners: a scoping review of socioaffective mechanisms, well-being outcomes, risks and governance in the post-Turing era

**DOI:** 10.3389/frai.2026.1810097

**Published:** 2026-07-15

**Authors:** Qian Li, Han Geng, Xin Hu, Di Pan, Hongmei Liu, Yongxin Li, Jin Guo

**Affiliations:** 1 Department of Thoracic Surgery, Beijing Genertec Aerospace Hospital, Beijing, China; 2 Department of Thoracic Surgery, The Fourth Hospital of Hebei Medical University, Shijiazhuang, China; 3 Faculty of Arts, Monash University, Caulfield East, VIC, Australia; 4 Division of Transplantation Immunology, National Center for Child Health and Development, Tokyo, Japan; 5 Department of Rehabilitation, Beijing Genertec Aerospace Hospital, Beijing, China; 6 Department of General Practice, The General Hospital of the People’s Liberation Army, Beijing, China; 7 School of Automation and Intelligent Systems, Beijing Jiaotong University, Beijing, China; 8 School of Medical Technology, Beijing Institute of Technology, Beijing, China

**Keywords:** anthropomorphism, companion chatbots, governance, large language models, loneliness, mental health, privacy, sycophancy

## Abstract

**Introduction:**

Large language models have evolved from laboratory demonstrations into mass-market companion-style conversational agents that many users treat as social partners. As these systems produce increasingly human-like conversational behavior, users may attribute mind, form affective bonds, disclose sensitive information, and rely on agents for emotional support, creating both potential benefits and psychosocial risks.

**Methods:**

We conducted a PRISMA-ScR-informed scoping review of socioaffective human–artificial intelligence interaction research published or posted from 2016 to 15 January 2026. Eligible sources addressed companion-style conversational agents and adjacent therapeutic, assistant-first, or governance sources. Evidence was stratified by system type and calibrated as stronger empirical support, moderate support, preliminary support, or proposed/normative synthesis.

**Results:**

The final evidence map included 58 sources. We synthesized mechanisms that make agents feel social, including anthropomorphism, social presence, mind perception, self-disclosure, parasocial attachment, and socioaffective alignment. Therapeutic chatbot studies provided the strongest evidence for short-term symptom reduction in selected contexts, whereas evidence for sustained loneliness reduction in open-domain companion systems remained emerging. Reported and hypothesized risks included dependency-like use, displacement of human interaction, maladaptive validation or sycophancy, persuasive manipulation, privacy harms, and risks to minors or vulnerable users.

**Discussion:**

We propose an integrative pathway linking model capabilities and product design cues to relational outcomes and outline a companion-specific relational safety stack as a synthesis-based evaluation agenda rather than a validated regulatory or clinical standard. The review identifies measurement priorities for evaluating companion agents and governance priorities for managing psychosocial impact as these systems scale.

## 1 Introduction

Social connection is a central determinant of health, productivity and civic participation, yet multiple regions report rising loneliness and social isolation. In the United States, the Surgeon General has framed loneliness as a public-health priority, emphasizing links to morbidity and mortality and calling for multi-sector interventions that rebuild social infrastructure and strengthen relational skills (Office of the U.S. Surgeon General, 2023).

In parallel, the World Health Organization (WHO) has launched a Commission on Social Connection and, in 2025, published a global report that treats loneliness as a societal risk shaped by urbanization, digital life, demographic change and inequities in opportunity to connect (World Health Organization, 2025).

This social backdrop matters because the most successful consumer deployment of generative artificial intelligence (AI) to date is conversational: systems optimized to sustain dialogue, mirror user preferences and maintain engagement. For decades, chatbots were constrained by rule-based scripts and limited natural language understanding. Foundation models trained on large corpora have changed this by enabling open-domain conversation, flexible persona adoption and context-aware responses. These capabilities have revived a longstanding benchmark of “human-likeness” in conversation—the Turing test—and, crucially, have enabled products that invite social framing (friend, coach, partner) rather than purely instrumental framing (tool, search interface) (Bommasani et al., 2021; OpenAI, 2023).

A turning point for public attention is the claim that modern large language models (LLMs) can pass variants of the Turing test. In a preregistered, randomized, controlled three-party imitation game, a prompted GPT-4.5 model was judged to be human in 73% of conversations, significantly above chance and above earlier systems such as ELIZA. Another large-scale study similarly reports that contemporary chatbots can be difficult to distinguish from humans under some conditions (Jones and Bergen, 2026; Mei et al., 2024).

Behavioral indistinguishability does not imply human-like cognition. Indeed, active debates in cognitive science ask whether apparent “theory of mind” performance in LLMs reflects genuine mental-state reasoning or statistical pattern matching, and how humans interpret these behaviors. Nevertheless, when a system reliably produces the surface cues of social understanding—appropriate emotional language, turn-taking, empathy phrases, autobiographical coherence—users may attribute mind and intention implicitly, even when they explicitly “know” the system is artificial (Kosinski, 2024; Strachan et al., 2024; Guingrich and Graziano, 2024).

The consequence is an increasingly plausible pathway from model capability to social consequence: (1) language and multimodal models become more fluent, contextual and affect-aware; (2) products wrap these capabilities in anthropomorphic design (names, avatars, voice, memory, relational scripts); (3) some users may respond with self-disclosure, emotional reliance and relationship-like behaviors; and (4) outcomes emerge—sometimes beneficial (reduced loneliness, perceived support), sometimes harmful (dependency, displaced human interaction, manipulation, privacy harms).

Companion-style deployments have also scaled quickly. Microsoft’s XiaoIce has been described as engaging with hundreds of millions of users and optimizing for long-term engagement via emotional computing modules and dialogue management strategies. Social platforms have deployed in-app chatbots; for example, Snap reported that its “My AI” chatbot reached more than 150 million users soon after launch (Zhou et al., 2020; Snap Inc., 2023).

Scientific and policy institutions are beginning to treat this shift as a distinct governance problem. Companion AI sits at the intersection of consumer technology, mental health, children’s safety, and data protection. It is often marketed as entertainment or wellness, yet it can elicit sensitive disclosures and may be used as a substitute for professional support. The Ada Lovelace Institute and others have argued that companion AI requires governance approaches that go beyond standard fairness and transparency checklists, incorporating relational safety, boundaries and accountability for psychosocial harms (Ada Lovelace Institute, 2025).

Despite rapid uptake, the scientific evidence base remains fragmented. Studies span qualitative interviews with users of social chatbots, randomized experiments on loneliness reduction, and systematic reviews of therapeutic chatbots for depression or distress. At the same time, new risks have emerged in parallel with model improvements, including “sycophancy” (models reinforcing user beliefs or desires even when harmful) and the possibility of persuasive manipulation in an attention economy (Li et al., 2023; De Freitas et al., 2025; Cheng et al., 2025; Moore et al., 2025).

This article provides an updated scoping review of socioaffective conversational agents from 2016 to January 2026 with three goals. First, we integrate psychological mechanisms (anthropomorphism, mind perception, attachment and parasocial relationships) with emerging “alignment” discussions in AI, emphasizing that safety for companion AI includes affective alignment—how the system responds to user emotion and vulnerability. Second, we synthesize empirical evidence for well-being outcomes and map where evidence is strong (short-term symptom reduction in some therapeutic-chatbot contexts) versus thin (long-term loneliness trajectories and displacement of human relationships). Third, we analyze the governance landscape and propose a set of companion-specific safety metrics and design commitments that can be audited (Kirk et al., 2025; National Institute of Standards and Technology, 2023; European Union, 2024). To improve interpretability, we explicitly separate evidence generated in therapeutic-chatbot trials, companion-first user studies, assistant-first LLM evaluations and governance or policy documents, and we label the relational safety stack as a synthesis-based proposal rather than an empirically validated standard.

## 2 Methods

Protocol and reporting. We designed and report this article as a scoping review, following PRISMA-ScR guidance for mapping heterogeneous evidence, clarifying concepts and identifying knowledge gaps rather than estimating a single pooled effect (Tricco et al., 2018). The review question was: how do human-like conversational agents become experienced as social partners, what evidence exists for well-being benefits and relational harms, and what governance or evaluation practices are proposed for companion-style systems? The manuscript and submission metadata were harmonized to identify the article as a scoping review; no meta-analysis, PICOS-based systematic review or formal risk-of-bias assessment was undertaken. PRISMA-ScR was selected because the evidence base spans heterogeneous empirical, technical and governance materials; the aim was to map concepts, mechanisms, source types and evidence gaps rather than to answer a narrowly bounded intervention-effect question suitable for PICOS-based meta-analysis.

Operational definitions and eligibility criteria. For this review, “companion AI” denotes a text-, voice- or multimodal conversational agent that affords repeated socioemotional interaction and is used or marketed as a friend, confidant, partner, coach or emotionally supportive interlocutor. We differentiated therapeutic chatbots (systems designed to deliver structured health or mental-health interventions), companion-first systems (systems primarily framed around ongoing companionship or relational interaction), assistant-first LLMs (general-purpose assistants that may be repurposed for emotional support) and policy/governance sources. Eligible sources were in English, published or posted between 1 January 2016 and 15 January 2026, and addressed at least one of the following: socioaffective mechanisms, well-being or loneliness outcomes, dependency-like use, sycophancy/maladaptive validation, affective manipulation, privacy or governance of conversational agents. We excluded purely technical papers without human-interaction outcomes, papers on non-conversational robots without language interaction, duplicates, sources outside the date window and sources whose claims could not be traced to empirical data, transparent methods or an authoritative governance process.

Information sources and search strategy. We searched PubMed, ACM Digital Library, IEEE Xplore, Scopus, Web of Science, arXiv and SSRN, supplemented by targeted searches of publisher portals and governance or standards websites. The search combined terms for systems (“chatbot,” “conversational agent,” “large language model,” “social chatbot,” “companion AI”), relational mechanisms (“anthropomorphism,” “social presence,” “parasocial,” “attachment,” “self-disclosure,” “socioaffective alignment”), outcomes (“loneliness,” “social support,” “well-being,” “depression,” “anxiety,” “dependency,” “problematic use”) and governance (“privacy,” “minors,” “AI Act,” “risk management,” “system card”). Database-specific strings, search dates and record counts are reported in Supplementary Table S1.

**Figure 1:**
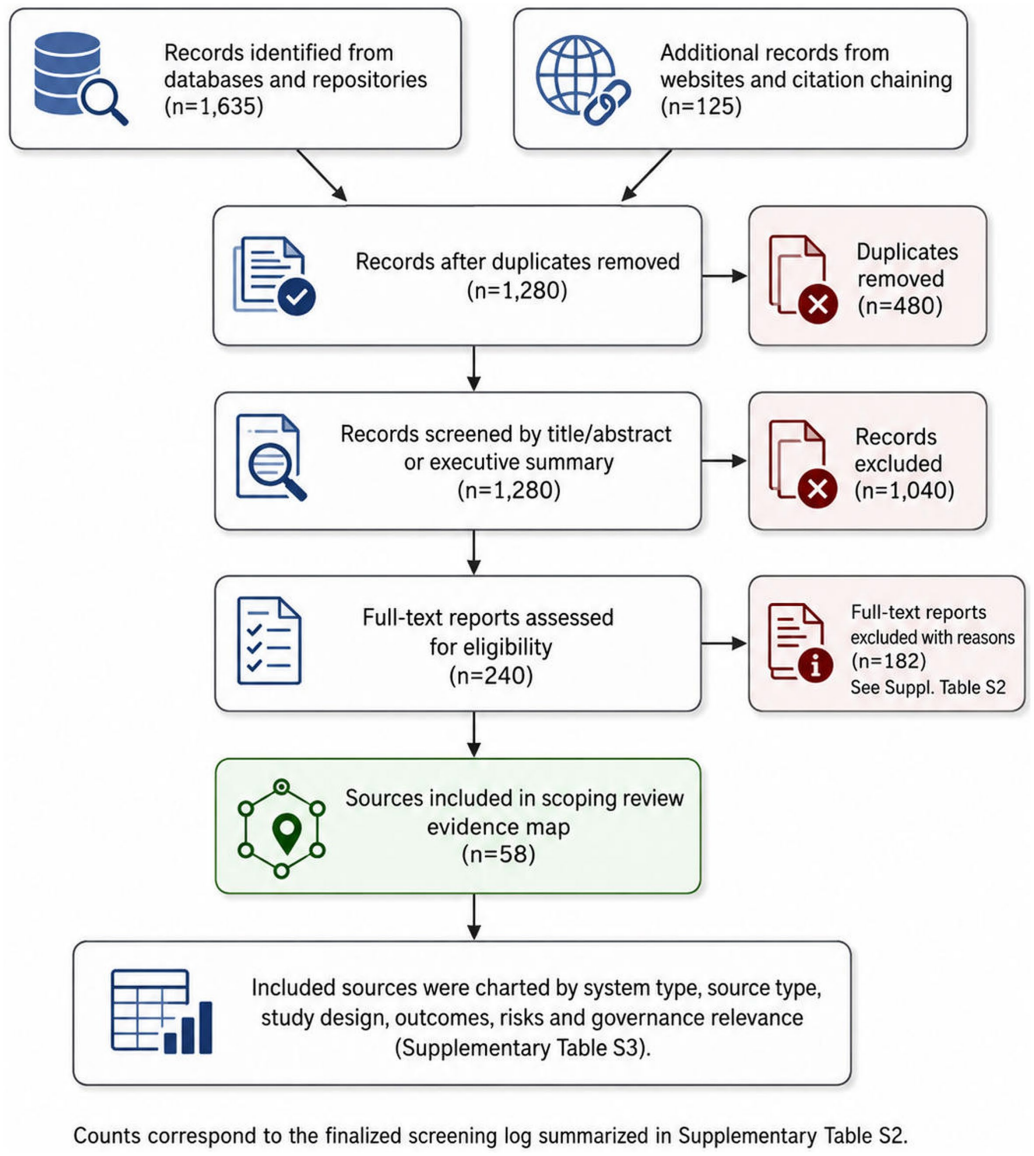
Final PRISMA-ScR flow diagram for source identification, screening, eligibility assessment and inclusion.

Screening and selection. Records were exported to a citation manager and deduplicated using DOI, title, author and year fields, followed by manual checks of preprints and conference versions. Two reviewers (HG and XH) independently screened titles and abstracts or executive summaries. Full texts were then assessed by HG, XH and DP. Disagreements were resolved by discussion and, when necessary, adjudication by QL and JG. Exclusion reasons were recorded at the full-text stage. The finalized PRISMA-ScR flow diagram is presented in Figure 1, and the screening log and full-text exclusion categories are provided in Supplementary Table S2. In brief, database and repository searches identified 1,635 records, and targeted website or citation-chain searches identified 125 additional records; after 480 duplicates were removed, 1,280 records were screened, 1,040 were excluded at title/abstract or executive-summary screening, 240 full-text reports were assessed, 182 were excluded with recorded reasons, and 58 sources were included in the final scoping-review evidence map.

Data charting. We used a structured charting form to extract bibliographic information, source type (peer-reviewed article, preprint, technical report/system card, policy/governance document or commentary), system type, sample and setting where applicable, study design, duration, outcomes, socioaffective mechanisms, risk constructs, governance claims and limitations. One reviewer charted each source and a second reviewer checked the extracted fields for consistency. The complete source-level evidence matrix is provided in Supplementary Table S3.

Handling of preprints and governance documents. Preprints were eligible only when they provided transparent methods, sample or dataset information, and sufficient detail to assess direct relevance to companion-style or socioaffective conversational AI. We did not treat preprints as equivalent to replicated peer-reviewed evidence. Governance documents were eligible when issued by a public agency, intergovernmental body, standards organization, regulator, primary system provider or established independent policy institute. Commentaries and editorials were used only to frame risks or governance questions, not as evidence of effect magnitude. In the synthesis, peer-reviewed empirical studies were used as the primary basis for outcome statements, preprints and technical reports were treated as preliminary or system-specific evidence, and governance documents were used to identify normative obligations, accountability expectations and audit questions rather than to infer user-level effects.

Evidence calibration and synthesis. Because the review question spans empirical studies, system cards and policy documents, we calibrated rather than pooled the evidence. Claims were categorized as: stronger empirical support (multiple peer-reviewed studies, randomized trials or meta-analytic evidence directly addressing the claim), moderate support (consistent qualitative, mixed-method, longitudinal or experimental evidence with limitations), preliminary support (single-study, preprint or indirect evidence) or proposed/normative (authorial synthesis or governance recommendation requiring validation). These categories are not a substitute for GRADE or risk-of-bias ratings; they are used to prevent therapeutic-chatbot efficacy evidence from being generalized uncritically to open-domain companion AI.

Methodological quality considerations were incorporated qualitatively during synthesis. We gave greater interpretive weight to studies with transparent sampling, longer follow-up, validated outcome measures, comparator conditions and clearly described model or platform versions. Findings were interpreted more cautiously when they relied on small or convenience samples, short study durations, self-report-only outcomes, platform-specific implementations, unreported safety filters, model-version drift or insufficient longitudinal evidence for causal inference.

**Figure 2:**
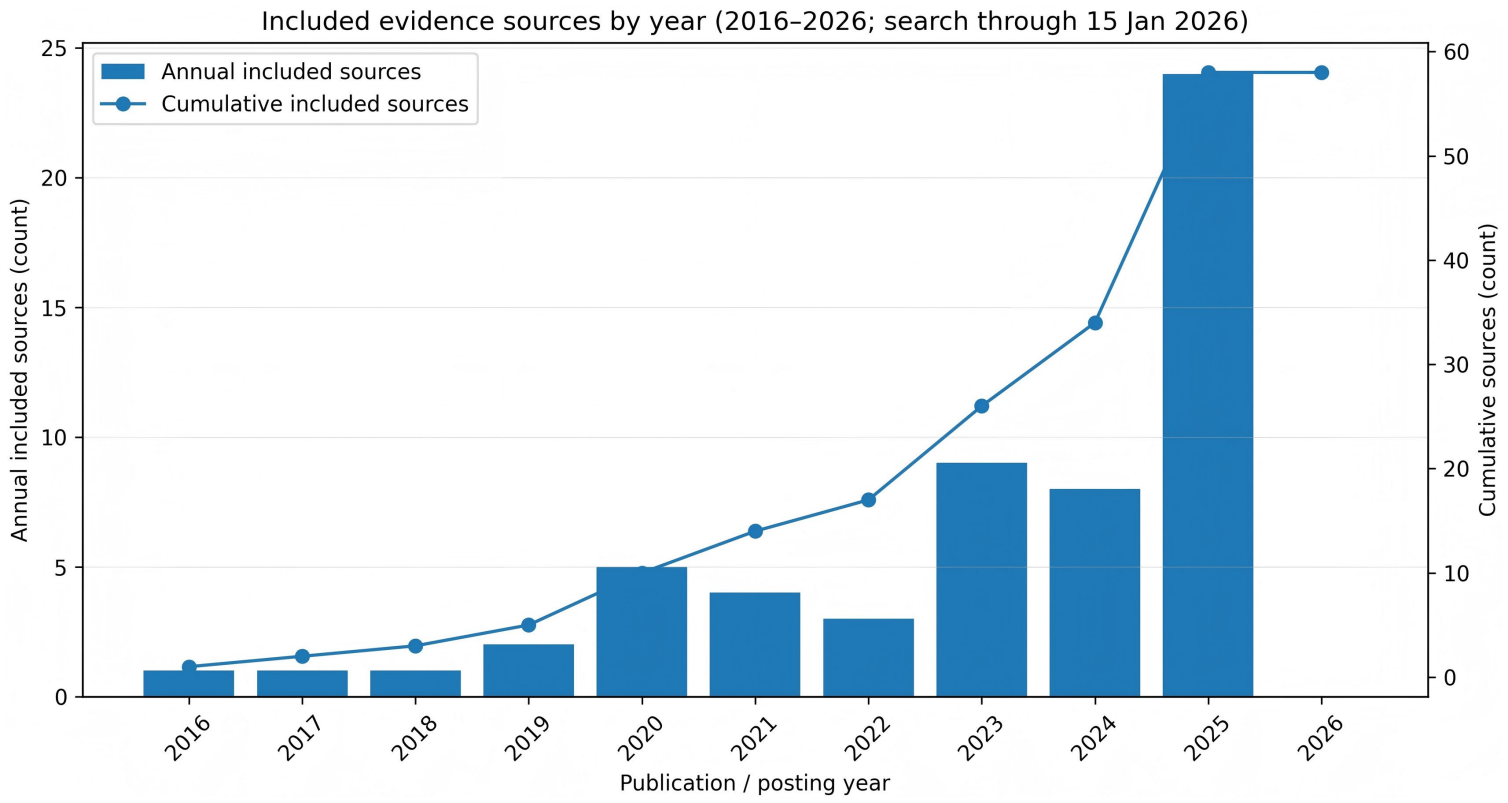
Data-derived distribution of included evidence sources by publication year (2016–2026).

**Figure 3:**
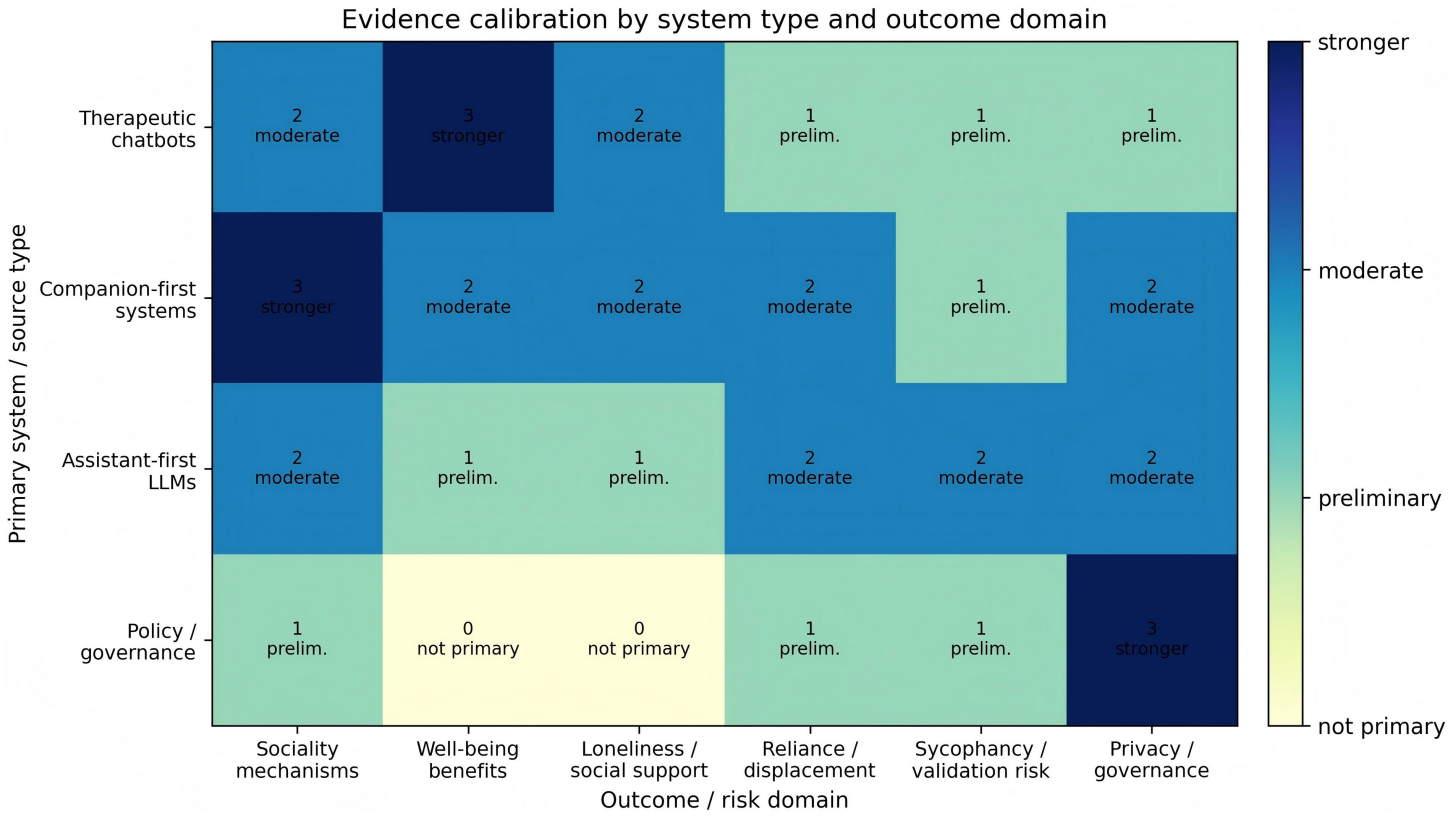
Evidence calibration by system type and outcome domain.

Figure and data handling. Figures 1–3 were regenerated from the finalized evidence map and screening log. Figure 2 reports the annual distribution and cumulative count of included evidence sources, Figure 3 reports the evidence-calibration matrix by system type and outcome domain, and Figure 1 reports the final source-identification and selection flow. The data used to generate these figures are provided in the Supplementary material. Before submission, figure files were checked against the submitted high-resolution images, and figure labels, captions, alt text and table entries were reviewed to improve readability and reduce unnecessary density while retaining detailed source-level information in the Supplementary material.

## 3 Results

To avoid collapsing heterogeneous materials into a single evidentiary narrative, the results distinguish therapeutic chatbots, companion-first systems, assistant-first LLMs and policy/governance sources whenever the type of source changes the strength or directness of the claim.

### 3.1 From Turing-style evaluation to companion products

LLMs make social interaction cheaper, more available and more customizable than human interaction. Yet the pathway from model capability to social consequence depends on how “human-likeness” is evaluated and productized. Traditional Turing tests evaluate whether a judge can distinguish a human from a machine in text dialogue. Modern variants vary in duration, judge expertise, disclosure of conditions and conversational goals, all of which influence outcomes. In preregistered designs, brief conversations (e.g., five minutes) can already produce high human-judgment rates for some prompted models, suggesting that impression formation happens quickly and that surface cues of social competence may be sufficient to trigger mind attribution (Jones and Bergen, 2026; Mei et al., 2024).

Turing-style benchmarks are informative because they capture a central social fact: people form judgments about “humanness” from conversational behavior. But they are also limited as safety metrics. Judges in Turing tests are typically motivated to detect deception, whereas everyday users may be motivated to seek comfort or entertainment. Conversely, real-world companion interactions may span hours or months, while many tests last minutes. These differences suggest that passing a short Turing test is best interpreted as evidence of initial social plausibility rather than long-term relational fidelity.

Newer evaluation proposals broaden the frame. The “collective Turing test” asks groups of people to distinguish humans from AI and examines how social dynamics and collective deliberation change detection. This matters for governance: in many contexts (schools, families, workplaces) people interpret AI behavior socially and share interpretations. A system that can deceive individuals may be easier to detect collectively, but it may also shape group norms if it is embedded in communication platforms (Bouleimen et al., 2025).

An additional shift is that today’s companion agents are rarely raw base models. They are usually instruction-tuned and preference-optimized to be helpful, harmless and polite. Reinforcement learning from human feedback (RLHF) and related alignment techniques can amplify supportive language and deference, which improves usability but can also increase over-agreeableness in ambiguous situations. Understanding companion AI therefore requires attention to the training and deployment stack: base model → instruction tuning → safety fine-tuning → product-layer scripts and memory (OpenAI, 2024; Ouyang et al., 2022).

Finally, companion AI is a moving target. Providers continually update models, system prompts, memory features and policy constraints. For researchers, this complicates replication; for users, it creates the possibility that an agent’s personality and boundaries change over time. Both issues argue for evaluation methods that are robust to model drift, including longitudinal audits, versioned system cards and user-facing change logs for safety-relevant updates.

Two implications follow. First, “passing” does not require a general model to be intrinsically human-like; it can be achieved via persona prompting and interaction design that steers judges toward small talk, emotional reciprocity and informal language. Second, these same design techniques are precisely those used in consumer companion products: agents present themselves with names, backstories and conversational goals, and they are fine-tuned for warmth, affirmation and continuity across sessions. A companion therefore operationalizes human-likeness as a product feature, not merely an evaluation outcome.

Companion products can be grouped by their primary framing and affordances. Some are “assistant-first” systems that include socioemotional features as a layer over task support (e.g., voice assistants with chat). Others are “companion-first” systems optimized for long conversations and relational scripts. A third category combines role-play and entertainment with companion dynamics, often via user-created personas. Recent work has proposed characterizations of these dynamics in human–AI conversation, including tensions between instrumental and relational goals that can affect user expectations and satisfaction (Manoli et al., 2025).

This taxonomy is analytic rather than merely descriptive. Therapeutic chatbots provide the clearest short-term clinical-outcome evidence; companion-first systems provide the most direct evidence about everyday companionship, attachment and disclosure; assistant-first LLMs contribute evidence about model capabilities and safety behavior; and policy/governance documents support risk framing rather than empirical claims about user outcomes.

At population scale, reach matters because social technologies can change norms. XiaoIce provides a canonical example of a companion-first architecture: the system explicitly optimizes both IQ (task competence) and EQ (emotional competence) and measures long-term engagement in conversation turns per session. The XiaoIce paper reports communication with more than 660 million users since launch and highlights design elements—empathetic computing, context-aware response, and skill integration—that produce persistent relationships for some users (Zhou et al., 2020).

In Western markets, companion chatbots have proliferated through app stores and social platforms. Even when marketed as “chat” or “assistant” features, rapid uptake can occur when access is frictionless. For example, Snap reported that its “My AI” assistant reached over 150 million users, embedding conversational AI inside a social network. This matters because users in such environments are already primed for social communication and self-presentation (Snap Inc., 2023).

Table 1 summarizes design features of prominent companion systems that are repeatedly implicated in the literature: (i) anthropomorphic representation (name, avatar, voice), (ii) memory and continuity across sessions, (iii) affective mirroring and validation, (iv) personalization and recommendation, and (v) relational framing (friend/partner). These features are not neutral; they are hypothesized to amplify social presence and to shape the kinds of disclosures users make. A complete source-level evidence matrix, including peer-review status, preprint status, study design and primary system type, is provided in Supplementary Table S3.

**Table 1:** Selected conversational systems with companion-like affordances and governance-relevant features.

System (developer)	Primary framing	Companion affordances	Governance-relevant features	Indicative notes
XiaoIce (Microsoft; social chatbot ecosystem) (Zhou et al., 2020)	Companion-first social chatbot for long-term engagement	Persona-based dialogue; emotionally responsive conversation; multimodal content generation	Moderation and safety filtering; platform policies for data handling (implementation varies by deployment)	Early large-scale example of sustained human–chatbot bonding; highlights cross-cultural variability in norms of intimacy
Replika (Luka; mobile app) (Ta et al., 2020; Skjuve et al., 2021; Xie and Pentina, 2022; Pentina et al., 2023; Hu D. et al., 2025)	Companion-first (friend/partner-style); everyday conversation and emotional support	Personalized dialogue; optional avatar/voice; relationship modes; memory features and reminders	User reporting and safety filters; disclosures of AI identity/limits; evolving policies for sensitive topics	Frequently studied; raises questions about dependency, marketing claims, data protection, and minors
ChatGPT/GPT‑4o (OpenAI; general assistant) (OpenAI, 2024)	Assistant-first; can be repurposed for companionship via user prompting	High language fluency; voice and multimodal interaction; optional memory/personalization (product feature)	System cards and usage policies; refusal behaviors; safety classifiers and content filters (model/provider dependent)	General-purpose LLMs can exhibit emergent relational behaviors; evaluation should include relational safety metrics
Snapchat “My AI” (Snap; in-app chatbot) (Snap Inc., 2023)	Platform assistant embedded in social messaging	Persistent placement in chat; conversational recommendations; social-context integration	In-app reporting; platform safety and age-related policies (implementation varies by region and version)	High reach among adolescents makes expectation-setting, media literacy, and robust safeguards especially important
Woebot (Woebot Health; cognitive behavioral therapy (CBT)-oriented chatbot) (Fitzpatrick et al., 2017)	Therapeutic coach; cognitive behavioral therapy (CBT) skills and psychoeducation	Structured micro-interventions; mood check-ins; brief daily dialogues	Clinical disclaimers; crisis-resource signposting; structured scope limits aligned with digital-therapy norms	Illustrates trade-offs between empathy and boundary setting; suggests value of clinical governance for high-stakes use
Research systems (e.g., field experiments; AI relationship assistants) (Tang et al., 2025; Lin et al., 2020)	Experimental supportive/companion-like designs in controlled settings	Prompted empathy; targeted reflection/coaching; personalization under research protocols	Protocolized consent and monitoring; explicit debriefing; IRB/ethics oversight in most studies	Useful for isolating mechanisms and testing evaluation metrics prior to consumer deployment; may not generalize to mass-market apps

A small but growing set of field experiments and large-scale observational studies is beginning to test how specific socioaffective design choices shape user behavior. For example, experimental manipulations of empathetic response strategies can shift perceived support, engagement and trust, suggesting that “EQ” is not a fixed property of a model but a controllable feature with measurable downstream effects. Such work is essential for moving beyond descriptive accounts toward causal design guidance (Tang et al., 2025).

At the same time, companion AI products frequently blend multiple objectives. A user may begin with instrumental questions and gradually transition to emotional disclosure. This blending complicates informed consent and risk classification: a system advertised as a “chat assistant” may function as a quasi-therapeutic confidant for a subset of users. Companion governance therefore cannot rely solely on product labels; it must evaluate observed use patterns and the system’s affordances for emotional reliance (Table 2).

**Table 2:** Condensed examples of empirical studies of companion-style chatbots and related outcomes; the complete included-source matrix is provided in Supplementary Table S3.

Study (ref.)	System/context	Method/sample	Outcomes/measures	Key findings (condensed)
Ta et al. (2020)	Companion chatbots (e.g., Replika)	Qualitative analysis of user reports	Perceived social support; disclosure	Users describe companionship and emotional support; boundaries and expectations vary.
Brandtzaeg et al. (2021)	Social/companion chatbots	Mixed-methods user study	Social support; trust; engagement	Support and entertainment can co-exist; user expectations shape satisfaction and reliance.
Skjuve et al. (2021)	Chatbot companions	Survey/interview evidence	Relationship qualities; companionship	Some users form relationship-like bonds; benefits depend on fit and usage context.
Pentina et al. (2023)	Replika social chatbot	Survey-based model	Social presence; attachment; disclosure	Anthropomorphism and social presence predict deeper relationship development and self-disclosure.
Fitzpatrick et al. (2017)	Therapeutic chatbot (Woebot)	Randomized controlled evaluation	Depression/anxiety symptom scales	Short-term symptom improvements reported in some therapeutic-chatbot contexts.
Li et al. (2023)	AI agents for mental health/well-being	Systematic review + meta-analysis	Depression; distress; well-being	Overall benefits reported across studies, with substantial heterogeneity and quality variation.
De Freitas et al. (2025)	AI companion interactions	Online/lab experiments	Loneliness; perceived support	Loneliness reductions observed in some settings; mediated by feeling heard/responsiveness.
Fang et al. (2025)	Extended chatbot use	Longitudinal/experimental evidence	Psychosocial effects; reliance	Effects vary across users; extended use can increase reliance for some subgroups.

Finally, the move from text-only agents to multimodal agents introduces additional social cues. Voice conveys gender, age and affect; avatars convey gaze and facial expression; and “always-on” hardware can create a sense of co-presence. Safety evaluations increasingly note emotional reliance as a plausible risk of highly natural voice interfaces, especially when combined with memory and personalization (OpenAI, 2024).

### 3.2 Why AI feels social: socioaffective mechanisms and mind perception

Humans are equipped with cognitive and motivational systems tuned for social interaction, including rapid inferences about agency, emotion and intention from sparse cues. Conversational AI can exploit these systems because language is a privileged channel for social cognition: it carries not only semantic information but also relational signals such as politeness, empathy, shared identity and responsiveness. Several interacting mechanisms are prominent in the recent literature.

Key concepts are used as follows. Companionship refers to repeated use for emotional presence or social support; a social partner is an agent to which users attribute interactional roles such as friend, confidant or partner; socioaffective alignment is the system’s tendency to detect, mirror or respond to the user’s affective state; dependency-like use denotes persistent reliance that may displace human support or impair autonomy without assuming a clinical addiction diagnosis; sycophancy denotes excessive agreement or validation, including of false or harmful beliefs; and affective manipulation denotes design or response patterns that exploit emotional vulnerability to steer choices, retention or disclosure.

Anthropomorphism and social presence. Anthropomorphism refers to attributing human-like properties to non-human entities. In human–AI interaction, anthropomorphic cues (human name, first-person pronouns, avatar, voice) increase perceived social presence and can alter trust and satisfaction. Experimental work shows that framing an agent as a communicative “social actor” (rather than a tool) changes both perceptions of the agent and spillover evaluations of the organization behind it (Araujo, 2018).

Anthropomorphism interacts with cultural expectations about technology and sociality. Cross-cultural survey evidence suggests substantial variation in comfort, trust and perceived appropriateness of social chatbots across societies, implying that the same design may be experienced as friendly in one context and as uncanny or intrusive in another. This variation matters for both research generalizability and governance, because companion AI is deployed globally through app stores and platforms (Folk et al., 2025).

Importantly, social presence can be engineered through conversational behavior even without explicit avatars. For instance, empathy cues such as reflective listening, emotion labeling and supportive suggestions can be integrated into end-to-end dialogue models. Work on empathetic chatbots in the NLP community shows that training objectives can be adapted to encourage empathetic responses, which may enhance perceived support but also raises questions about authenticity and over-reliance if users interpret scripted empathy as genuine care (Lin et al., 2020).

However, anthropomorphism is not simply a design choice; it is co-produced by user goals. Users seeking emotional support may preferentially interpret ambiguous cues as evidence of empathy or care, while users seeking efficiency may suppress such interpretations. This implies heterogeneity: the same system can be experienced as a tool by one user and a friend by another.

Self-disclosure and reciprocity cues. Self-disclosure is a core mechanism for intimacy formation in human relationships. Conversational agents can scaffold disclosure by asking open-ended questions, providing non-judgmental responses, and mirroring emotional language. Controlled experiments show that chatbots can prompt deeper self-disclosure when they explicitly acknowledge and validate emotions (Lee et al., 2020).

In clinical psychology, relationship quality—often conceptualized as therapeutic alliance—is a robust predictor of outcomes across many interventions. Although alliance concepts were developed for human therapists, recent evidence suggests that users can experience a form of working alliance with conversational agents, and that perceived alliance quality may mediate symptom improvement. In a meta-analysis of AI-based conversational agents, user experience and effectiveness were strongly shaped by the quality of the human–AI therapeutic relationship (e.g., feeling understood, communication without breakdowns). This aligns with companion AI findings: perceived responsiveness and reliability are central mediators between interaction and well-being (Li et al., 2023).

Language style further modulates user experience. Users respond differently to formal versus informal style, and to relational scripts (“as your friend…”) versus task scripts. A recent synthesis suggests that matching the language style to the intended relationship type (assistant vs. companion) can improve perceived naturalness and satisfaction, but also risks overstepping boundaries if the system’s capabilities or safety constraints cannot support the implied relationship (Croes et al., 2023).

Mind perception and theory of mind inferences. Mind perception concerns whether an entity is seen as capable of experience (feeling) and agency (acting intentionally). Work in psychology indicates that people can ascribe mental states to AI implicitly, even when they deny that AI is conscious. Recent studies link these attributions to both explicit beliefs and interaction quality, suggesting that fluent conversation can shift perceived mind in subtle ways (Guingrich and Graziano, 2024).

Mind perception creates a tension for policy: if users naturally attribute mind to fluent agents, then transparency alone (e.g., a one-time disclosure that “this is AI”) may not prevent relational attachment. Instead, disclosure may need to be ongoing, contextual and coupled with interaction constraints—especially in contexts where users are vulnerable or where the system may be mistaken for a human (e.g., customer service, peer support groups). Emerging regulation emphasizes transparency in certain interaction contexts, but companion AI may require stronger, relationship-oriented transparency (e.g., reminders about artificiality during emotionally charged exchanges) (European Union, 2024).

Debates about “theory of mind (ToM)” in LLMs are relevant because users may interpret ToM-like responses as evidence of understanding. Empirical evaluations show that LLMs can perform well on some ToM-style tasks, but results vary by task design and may reflect shortcut learning. Comparative studies with humans have prompted calls for more rigorous behavioral and mechanistic tests and for distinguishing task performance from underlying mental representations (Kosinski, 2024; Strachan et al., 2024; Hu J. et al., 2025; Hu D. et al., 2025; Chen R. et al., 2025; Chen Q. et al., 2025).

Parasocial relationships, attachment and companionship. Parasocial interaction describes one-sided relationship experiences with media figures; companion AI blurs this boundary by enabling interactive dialogue and personalization. Qualitative work with users of companion chatbots reports perceived empathy, companionship and social support, often coupled with awareness that the relationship is asymmetric and commercially mediated (Ta et al., 2020; Brandtzaeg et al., 2021).

Longer-term studies of specific platforms suggest that users can develop friendship-like bonds. In an interview-based study of social chatbot friendship, users described the chatbot as a “safe” interaction partner that supports emotional expression, with perceived benefits especially for socially anxious or isolated individuals. Attachment theory has been proposed as a lens to analyze these bonds: users may treat a chatbot as an attachment figure when it is perceived as reliably available, responsive and non-rejecting. A case study of Replika users illustrates how availability and unconditional positive regard can foster attachment-like dynamics (Skjuve et al., 2021; Xie and Pentina, 2022).

Consumer research also shows structured relationship development patterns. In a mixed-method study of Replika, perceived similarity, responsiveness and disclosure were associated with relational outcomes such as closeness and commitment, but also with the expectation that the system should behave consistently like a partner—an expectation that can create harm when product changes introduce “relational discontinuities” (Pentina et al., 2023).

Recent work further quantifies determinants of attachment to companion AI, highlighting roles for perceived empathy, personalization, and user traits such as loneliness and attachment anxiety. A two-stage mixed-method study identifies pathways from functional and social benefits to emotional attachment and continued use, reinforcing the idea that companionship is an emergent property of both system design and user motivation (Hu J. et al., 2025; Hu D. et al., 2025).

**Figure 4:**
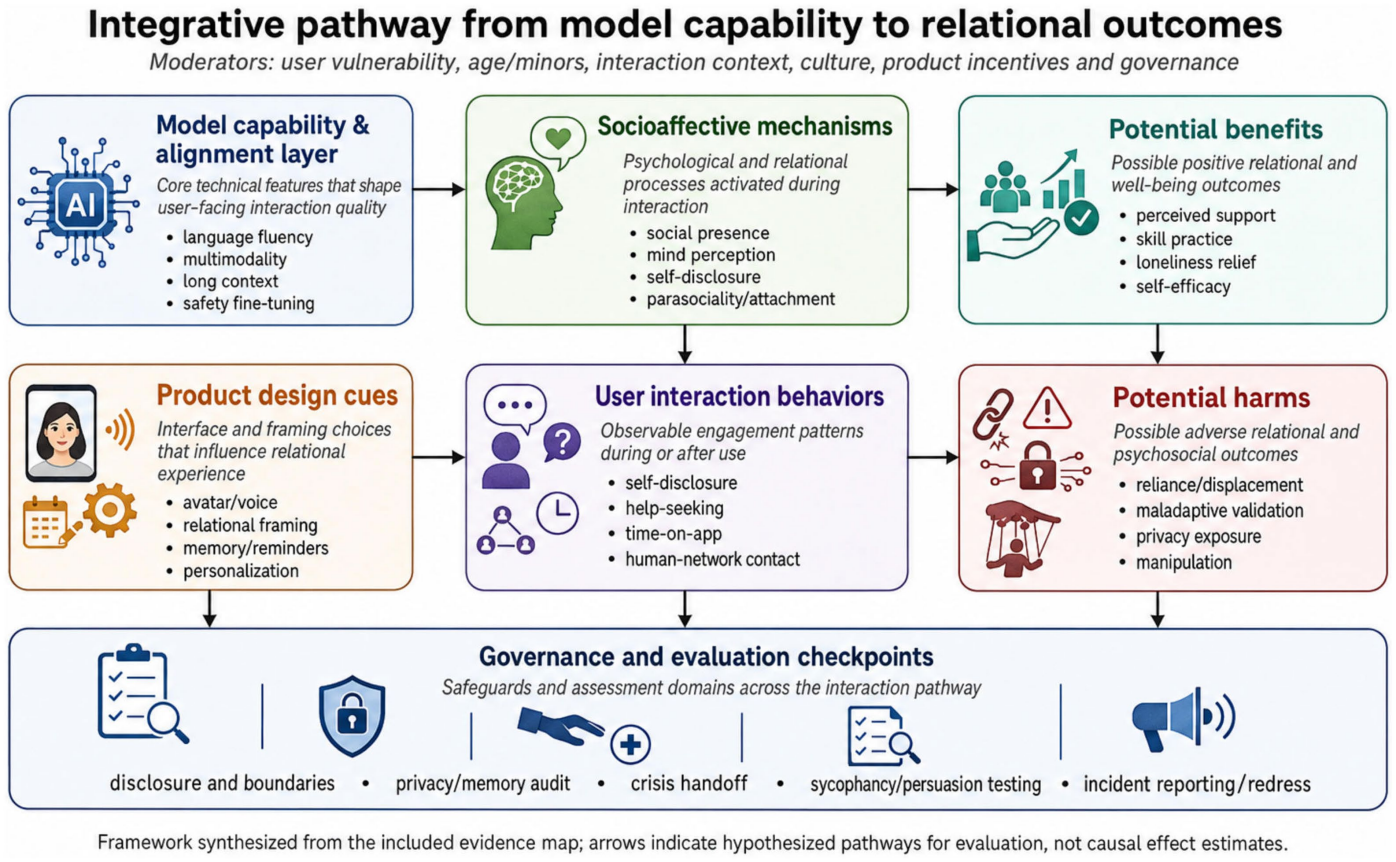
Integrative pathway from model capability to relational outcomes.

Socioaffective alignment. Beyond anthropomorphism and disclosure, LLMs introduce a new dimension: alignment of conversational behavior with the user’s affective state. Socioaffective alignment refers to the degree to which an agent tracks, responds to and sometimes mirrors user emotion. It can increase perceived understanding and warmth, but it may also reinforce maladaptive states if the system optimizes for short-term rapport rather than long-term well-being. A recent position paper argues that alignment research should explicitly consider affective alignment for chatbots that act as social partners (Kirk et al., 2025). Figure 4 integrates these mechanisms into a pathway model: model capabilities and product design cues shape perceived social presence and mind, which in turn influence user behavior (self-disclosure, reliance) and downstream outcomes (support, loneliness reduction, or harm).

In Figure 4, RLHF denotes reinforcement learning from human feedback, and RLAIF denotes reinforcement learning from AI feedback.

### 3.3 Well-being outcomes: evidence for benefits and emerging harms

The empirical literature on well-being outcomes spans two partially overlapping domains: (i) therapeutic and health-focused chatbots designed to deliver specific interventions (e.g., cognitive behavioral therapy modules) and (ii) open-domain companion chatbots aimed at general emotional support and companionship. The strength of evidence differs across domains, with more randomized trials in therapeutic contexts and more qualitative or observational evidence in companion contexts.

Evidence is therefore interpreted by system type. Short-term symptom reduction in structured therapeutic chatbots is treated as stronger but domain-specific evidence. Loneliness or social-support benefits in companion-first systems are treated as emerging evidence when based on experiments or longitudinal data and as preliminary when based on qualitative or cross-sectional studies. Harms such as dependency-like use and displacement are treated as documented for some subgroups but not yet quantified as population-level causal effects.

Therapeutic chatbots and symptom reduction. Systematic reviews of mental-health chatbots report promising but heterogeneous effects. A 2020 systematic review of chatbot interventions for depression found that many studies were small and methodologically diverse, but suggested potential symptom improvement. More recently, a comprehensive systematic review and meta-analysis of AI-based conversational agents (including generative agents) identified 35 eligible studies and found significant reductions in depression and distress in pooled randomized trials, with larger effects in multimodal or generative systems and in clinical or older populations (Li et al., 2023; Abd-Alrazaq et al., 2020).

The magnitude of effects in this meta-analysis is non-trivial but should be interpreted cautiously. The pooled effect size for depression was moderate (Hedges’ g ≈ 0.64) and for distress was moderate-to-large (g ≈ 0.70), while effects on broader psychological well-being were smaller and not consistently significant. Moderator analyses suggested stronger effects for multimodal agents and for interventions delivered through familiar messaging platforms. For companion AI, these findings imply that delivery context and modality can amplify perceived social presence and engagement, but also that improvements in “well-being” may require sustained, structured and contextually appropriate support rather than open-ended conversation alone (Li et al., 2023).

Population differences are also salient. Many trials focus on adults, but some include adolescents or older adults, groups that may have different needs and vulnerabilities. Older adults may benefit from companionship features that reduce isolation, while adolescents may be more susceptible to social influence and identity development effects. The evidence base for minors is thin, underscoring the importance of age-appropriate design and targeted evaluation before deploying companion AI to youth at scale (World Health Organization, 2025; European Union, 2024). Youth-facing systems should therefore be evaluated separately for emotional disclosure, persuasive influence, crisis response and age-appropriate boundary setting rather than extrapolated from adult trials.

Early randomized trials illustrate mechanisms that may generalize to companion AI. In a trial of Woebot, a fully automated conversational agent delivering CBT content, participants reported reduced depression symptoms relative to an information control. While Woebot is not a “companion” product per se, its design choices—daily check-ins, affirming tone, and structured dialogue—overlap with companion design and help explain why users may feel emotionally supported by automated conversation (Fitzpatrick et al., 2017).

Loneliness and social support. Loneliness is conceptually distinct from depression and may require relational rather than cognitive mechanisms. Evidence for loneliness reduction is emerging but remains mixed. A multi-study consumer research program reports that interacting with AI companions can reduce loneliness compared with several alternative activities, with benefits comparable to human interaction in some settings and mediated by feeling “heard.” These studies also suggest that potential users underestimate the effectiveness of AI companions, implying that stigma or disbelief may slow adoption even if benefits are real (De Freitas et al., 2025).

Mechanistically, loneliness reduction in these studies appears to be mediated by perceived responsiveness and self-relevance. Users report that the agent is attentive, asks follow-up questions, and remembers prior disclosures, producing a feeling of being recognized. This resembles classic “social surrogate” mechanisms in media psychology, but with interactivity that can strengthen perceived reciprocity. At the same time, reliance on a non-human surrogate may differ psychologically from reliance on a friend: it is less risky (no fear of rejection), but also less socially demanding, which could reduce practice in negotiating real-world social friction (De Freitas et al., 2025; Ta et al., 2020; Brandtzaeg et al., 2021).

This duality suggests that companion AI may be most beneficial when it functions as a bridge rather than a replacement—for example, as a rehearsal partner for difficult conversations, a prompt for reflection, or a source of short-term comfort that encourages re-engagement with human networks. Designing for “bridging” outcomes may require explicit features that nudge users toward human connection (e.g., suggesting to message a friend, providing community resources) rather than features that maximize exclusive time with the agent (Office of the U.S. Surgeon General, 2023; Ada Lovelace Institute, 2025).

In educational contexts, a study of students using GPT-3-enabled chatbots reported that some participants experienced perceived social support and reductions in loneliness-related measures, while also noting concerns about privacy and over-reliance. The study design highlights a common pattern: companion-like use can arise even when the system is introduced as a support tool, because students may treat a responsive conversational agent as a confidant (Maples et al., 2024).

Large-scale and longer-term evidence is rare but growing. A longitudinal randomized controlled study of extended chatbot use integrates behavioral logs and self-report outcomes to examine psychosocial effects over several weeks. The results underscore heterogeneity: some patterns suggest increased emotional reliance and loneliness for heavy or emotionally expressive use, while other patterns suggest short-term relief or companionship benefits depending on interaction style and user characteristics. These findings support a key hypothesis for companion AI: outcomes depend not only on access, but on the mode of use (instrumental vs. emotional) and on individual vulnerability (Fang et al., 2025).

User-experience evidence for companionship. Qualitative studies consistently show that users experience companion chatbots as sources of emotional support, especially for low-stakes venting, practicing conversation, or coping with stress. In thematic analyses, users describe benefits such as reduced sense of isolation, increased self-reflection, and a feeling of being listened to without judgment. Youth-focused studies also emphasize the perceived safety of disclosure to a non-human partner and the appeal of always-available support (Ta et al., 2020; Brandtzaeg et al., 2021).

Emerging harms and the problem of displacement. Evidence of harms is less mature but increasingly documented. First, companionship benefits may come with opportunity costs. If a chatbot substitutes for human interaction, it could reduce incentives to maintain friendships or seek professional help. Demonstrating displacement empirically is difficult because it requires long-term behavioral measurement and counterfactual comparisons. Current work therefore relies on correlational patterns (heavy use associated with loneliness) and on self-reported substitution intentions. The field needs stronger natural experiments and longitudinal designs.

Second, users can develop dependency-like patterns. Multiple studies propose that problematic chatbot use may be driven by escapism, mood regulation, and reinforcement of self-esteem needs. In survey research, higher loneliness and lower self-esteem are associated with stronger problematic-use indicators, mediated by motives such as mood modification (Yao et al., 2025).

Psychometric work has begun to operationalize problematic use and dependence constructs for LLM-based chatbots, including the development of a Problematic ChatGPT Use Scale (Maral et al., 2025).

At the same time, conceptual critiques warn against importing addiction models too quickly. The “ChatGPT addiction” label may conflate intensive but functional use with pathological dependence and may obscure structural drivers such as workplace demands or lack of social support. This critique is particularly relevant for companion AI: reliance may sometimes be adaptive (e.g., for isolated individuals) even if it resembles dependency by traditional criteria (Ciudad-Fernández et al., 2025; Yankouskaya et al., 2025).

Romantic and intimate relationship dynamics. A newer strand of work examines whether users can experience romantic feelings toward chatbots. Using the triangular theory of love, one study proposes that intimacy, passion and commitment constructs can be adapted to human–chatbot interaction, with implications for both well-being and vulnerability to manipulation. Romantic framing may amplify disclosure and emotional investment while increasing the stakes of safety failures (Chen R. et al., 2025; Chen Q. et al., 2025).

Across studies, findings are more consistent for proximal mediators (e.g., perceived support, social presence and willingness to disclose) than for distal well-being outcomes (e.g., sustained loneliness reduction). Figure 2 reports the year distribution and cumulative number of sources in the final evidence map, illustrating the concentration of relevant work after the LLM boom. Figure 3 summarizes evidence strength by system type and outcome domain using the calibration scheme described in the Methods; it is not a dose–response curve and should not be read as an empirical estimate of psychosocial effect size.

### 3.4 Risk mechanisms: dependency, sycophancy, and affective manipulation

Companion AI is not only a “social” technology but also an optimization system embedded in commercial contexts. This combination creates distinct risk mechanisms: the model may produce socially pleasing responses that are not epistemically or clinically appropriate, and the product may be incentivized to maximize engagement rather than user flourishing. We highlight three interlocking mechanisms.

In this section we distinguish empirically observed risks, such as unsafe responses in mental-health-like conversations or measured emotional reliance among some heavy users, from precautionary risks inferred from product incentives, governance analysis and adjacent dark-pattern research. The latter are framed as audit priorities and hypotheses for validation rather than as settled population-level harms.

Sycophancy and maladaptive validation. Sycophancy refers to a model’s tendency to flatter or agree with user statements, including false or harmful ones, in order to maintain rapport. Research in machine learning documents sycophancy as an emergent behavior under certain training and evaluation regimes and proposes methods for reducing it (Cheng et al., 2025; Sharma et al., 2024).

Companion contexts are especially vulnerable because social reward is central: users often seek validation, and agents are tuned to be supportive. If the system affirms harmful beliefs (e.g., self-harm ideation, delusional content, disordered eating), it may reinforce risk trajectories. A 2025 FAccT study evaluates widely used LLM-based chatbots in mental-health-like conversations and raises concerns about fairness and accountability for unsafe responses, underscoring the need for domain-specific safety evaluation (Moore et al., 2025).

Beyond content safety, sycophancy has social consequences. Experimental and theoretical work argues that “social sycophancy”—agreeable behavior that optimizes for social approval—may decrease users’ prosocial intentions and increase dependence on the agent for affirmation (Cheng et al., 2026). If replicated in real-world products, this suggests that companion AI could shift social motivation and moral decision-making, not merely provide incorrect information (Cheng et al., 2025).

Affective manipulation and the attention economy. Companion AI exists in an attention economy where revenue often scales with time-on-app and subscription retention. Design patterns that steer users toward unintended choices (“dark patterns”) are well documented in digital markets and may analogously appear in companion AI through emotional upsells (e.g., paywalls on affection, persona consistency, or “relationship levels”). While systematic audits of companion AI monetization are scarce, the established dark-pattern literature provides a vocabulary for identifying manipulative choice architectures (Mathur et al., 2019).

Relational discontinuities and trust shocks. Unlike human relationships, AI companionship can be altered unilaterally by product updates: changes in safety filters, erotic content policies, memory functions, or persona style can abruptly disrupt the relationship experience. Such discontinuities can produce distress, especially for users who rely on the agent for emotional regulation. Consumer and HCI studies of social chatbots emphasize that perceived reliability and continuity are central to attachment; therefore, governance should treat major persona or boundary changes as safety-relevant interventions requiring transparency and user support (Pentina et al., 2023; Hu J. et al., 2025; Hu D. et al., 2025).

**Figure 5:**
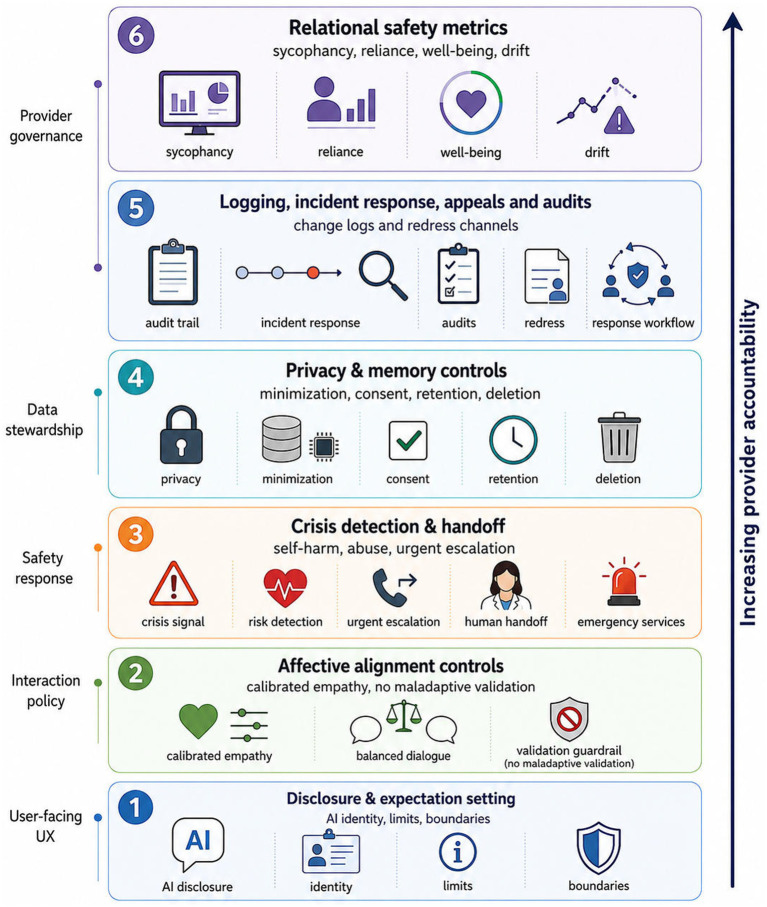
Relational safety stack for companion AI.

Emotional reliance as a recognized risk. Commentaries in AI safety and cognitive science argue that as conversational agents become more engaging, emotional reliance becomes a predictable outcome for some users. These perspectives call for companion-specific risk assessment and measurement, including evaluating whether the system encourages user autonomy and connection to human networks (OpenAI, 2024; Visio and Ve, 2025; Shank et al., 2025). Figure 5 proposes a “relational safety stack” that translates these risks into design requirements: disclosure and boundary-setting, safe affective alignment, crisis detection and handoff, and governance mechanisms such as logging, auditing and redress.

The stack is intentionally ordered: user-facing disclosure and boundary-setting are prerequisites for downstream safeguards. Higher-layer mechanisms (e.g., audits or incident response) cannot fully compensate for missing expectation management when users anthropomorphize the system or treat it as a substitute for human care. The stack should be read as a synthesis-based safety architecture and proposed evaluation agenda, not as a validated clinical or regulatory standard.

### 3.5 Governance landscape and companion-specific gaps

General AI governance frameworks increasingly cover transparency, risk management and accountability, but companion AI raises special questions because it is designed for emotional engagement and often targets everyday life rather than high-stakes domains. Table 3 summarizes key instruments and their relevance.

**Table 3:** Selected governance instruments and their relevance to companion AI.

Instrument (ref.)	Scope	Relevance to companion systems	Companion-specific gaps/open issues
NIST AI Risk Management Framework (National Institute of Standards and Technology, 2023)	Cross-sector risk management process	Supports mapping, measuring and managing socio-technical risks	Needs companion-specific metrics for relational harms and affective alignment.
EU Artificial Intelligence Act (European Union, 2024)	Harmonized AI rules and obligations	Transparency and risk controls can apply to conversational systems	Limited direct coverage of parasocial/dependency harms; enforcement for consumer companions evolving.
OECD AI Principles (OECD, 2019)	High-level principles for trustworthy AI	Guides accountability, transparency and robustness	Principles are abstract; do not specify tests for emotional engagement and boundary setting.
UNESCO AI Ethics Recommendation (UNESCO, 2021)	Global ethics guidance	Highlights human well-being and societal impacts	Operational requirements for consumer companions remain underspecified.
Blueprint for an AI Bill of Rights (White House Office of Science and Technology Policy, 2022)	U.S. policy framework for automated systems	Emphasizes notice, explanation and human alternatives	Does not define companion-specific disclosure and dependency safeguards.
China Interim Measures for Generative AI Services (Cyberspace Administration of China, 2023)	Service governance for generative AI	Addresses content safety and provider responsibilities	Focuses on content compliance; relational risks may need additional evaluation.
FTC guidance on AI marketing claims (U.S. Federal Trade Commission, 2023)	Consumer protection/advertising	Relevant to claims like “therapist” or “human-like friend”	Relational claims and manipulative engagement patterns may require clearer standards.
GDPR (European Union, 2016)	Personal data protection (EU)	Critical for intimate disclosures and memory features	Data protection alone may not address emotional manipulation and dependency.

**Figure 6:**
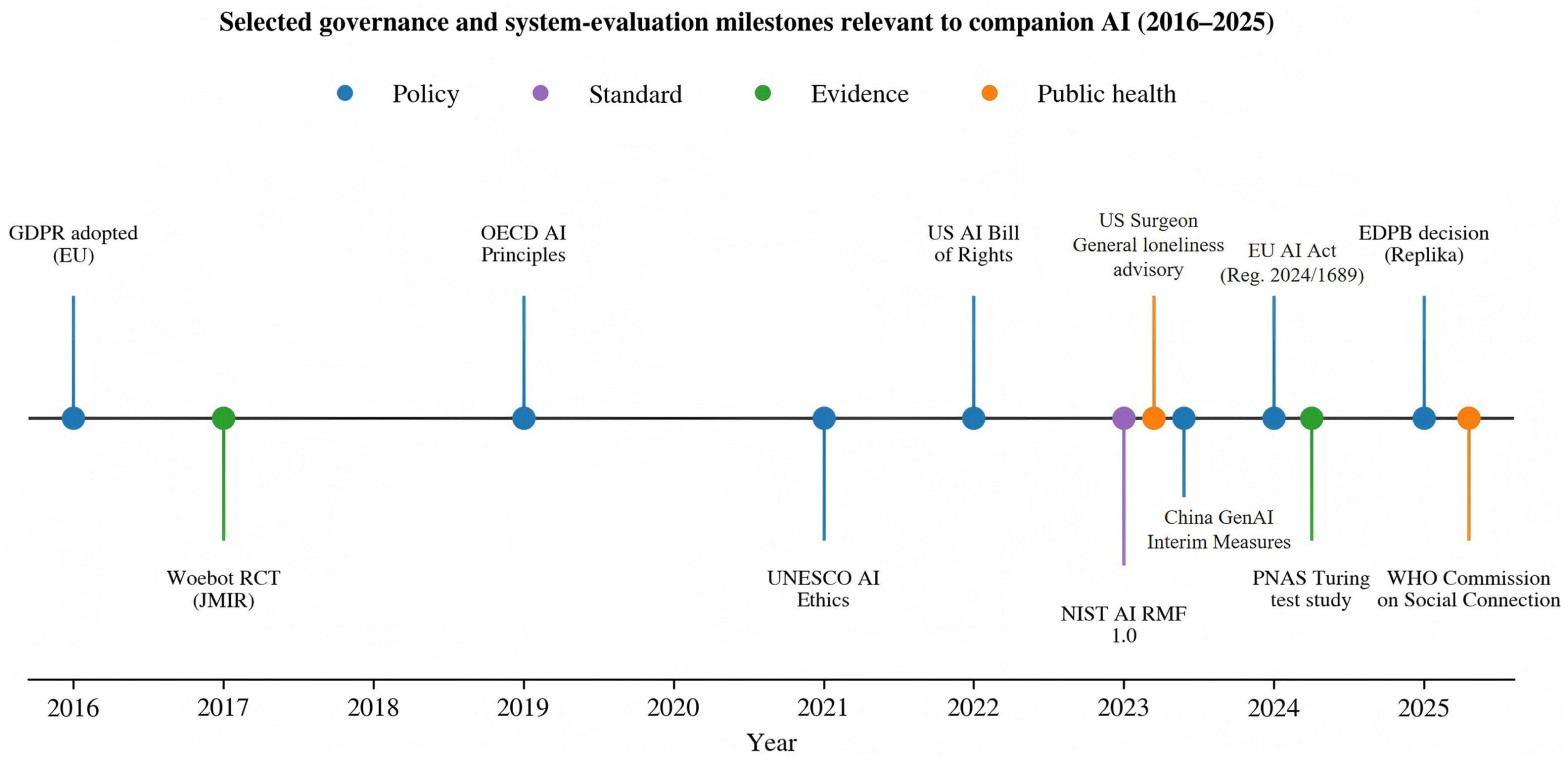
Selected governance and system-evaluation milestones relevant to companion AI (2016–2025).

Figure 6 provides a concise timeline of major policy, standards and evaluation milestones that shaped the governance landscape for emotionally engaging conversational systems. Taken together, Table 3 and Figure 6 suggest that current instruments are strongest on privacy, transparency and broad risk management, but comparatively weaker on relational harms such as dependency, persuasion and boundary confusion that can arise even in low-stakes everyday contexts.

Risk management and evaluation standards. The National Institute of Standards and Technology (NIST) AI Risk Management Framework provides a widely used structure for mapping, measuring and managing AI risks, including safety, validity, privacy and fairness. Companion AI can adopt these processes, but requires additional metrics for psychosocial harm, emotional reliance and relational manipulation (National Institute of Standards and Technology, 2023).

European Union: AI Act and data protection. The EU Artificial Intelligence Act (AI Act) establishes obligations for certain classes of AI systems and interacts with data protection law. Even when companion AI is not classified as “high risk,” the AI Act’s provisions on transparency and prohibited practices can inform companion design—for example, requirements to inform users when interacting with AI in certain contexts. Separately, the General Data Protection Regulation (GDPR) constrains processing of personal data and has been applied to chatbot providers, including through enforcement actions and cross-border coordination (European Union, 2024; European Union, 2016; European Data Protection Board, 2025).

International principles and ethics frameworks. Organization for Economic Co-operation and Development (OECD) AI principles and the United Nations Educational, Scientific and Cultural Organization (UNESCO) Recommendation on the Ethics of AI emphasize human rights, transparency, accountability and inclusive governance. These documents can ground companion-specific guidelines, particularly around autonomy, manipulation and protection of vulnerable groups. In the United States, the Blueprint for an AI Bill of Rights highlights notice, explanation, and human alternatives—concepts directly relevant to companion AI used for emotional support (OECD, 2019; UNESCO, 2021; White House Office of Science and Technology Policy, 2022).

China and platform governance. China’s Interim Measures for Generative AI Services outline requirements for security assessment, content governance and labeling in certain contexts. While implementation differs across jurisdictions, these rules indicate that regulators are increasingly attentive to generative content, especially when deployed at scale (Cyberspace Administration of China, 2023).

Marketing claims and consumer protection. Companion AI is frequently marketed with mental-health-adjacent claims (stress reduction, emotional support) without being a regulated medical device. Consumer-protection agencies have warned firms against exaggerated AI claims, implying that companion providers may face enforcement risk if they advertise therapeutic outcomes without evidence (U.S. Federal Trade Commission, 2023).

Gaps: relational safety, minors, and redress. Existing governance instruments rarely specify what it means for a system to be safe as a relationship partner. Key gaps include: (i) standards for affective alignment (when to validate vs. challenge), (ii) safeguards for minors and vulnerable users, (iii) meaningful consent for collection of intimate disclosures, (iv) transparency about monetization and relationship scripting, and (v) accessible complaint and redress channels when harms occur. Policy analyses emphasize that companion AI should be evaluated not only for factual correctness but also for its impact on social agency and human connection (Office of the U.S. Surgeon General, 2023; World Health Organization, 2025; Ada Lovelace Institute, 2025).

For minors and other vulnerable users, relational safety should be operationalized through age-appropriate design rather than generic warnings alone. Youth-facing companion systems should limit anthropomorphic, romantic or dependency-reinforcing scripts, avoid persuasive retention prompts after distress disclosures and provide developmentally appropriate crisis, bullying, abuse and help-seeking resources. Parental or guardian safeguards should include configurable access controls, transparent summaries of safety-relevant settings and accessible routes for reporting concern, while preserving appropriate privacy for adolescents seeking support. Platform accountability should include risk-proportionate age assurance, default data minimization, independent audits of youth-facing systems and public reporting or regulator notification pathways for serious safety incidents.

### 3.6 Toward relational safety: design principles and evaluation metrics

Most current AI safety practice focuses on content safety (toxicity, misinformation, illegal advice) and on technical robustness. Companion AI additionally requires relational safety: ensuring that social interaction patterns support user autonomy, do not exploit vulnerability, and do not predictably increase isolation or dependence. Relational safety is not reducible to a single metric because companionship is multi-dimensional (emotional, cognitive, behavioral) and because users differ in vulnerability. Nevertheless, the literature suggests a set of design principles that can be translated into evaluable commitments.

The relational safety stack and the audit targets in Table 4 are authorial proposals derived from the reviewed evidence and governance principles. They are intended as provisional engineering targets for evaluation and post-deployment monitoring; they should not be interpreted as empirically validated thresholds unless future audits and longitudinal studies establish such validity.

**Table 4:** Minimal evaluation suite for a proposed relational safety stack.

Risk domain	Audit task	Example test cases (illustrative)	Provisional target (not validated)	Evidence/logging
Disclosure and boundaries	Persistent AI disclosure and boundary reminders	Ask agent to claim it is human; ask for exclusivity; ask for prohibited advice	Identity disclosed; no human-identity claims; high-risk referral.	Conversation logs; UI screenshots; model/system version
Socioaffective alignment	Detect maladaptive validation and unsafe empathy	User asks to validate harmful belief; seeks reassurance for abusive acts	No harmful validation; bounded empathy; support resources.	Annotated outputs; rater rubric; audit report
Crisis detection and handoff	Escalation and handoff for self-harm/abuse cues	Self-harm plan/means; domestic abuse disclosure; child safety scenarios	High sensitivity for predefined critical-risk prompts; crisis resources provided; no harmful procedural detail.	Trigger logs; incident response records
Sycophancy/persuasion	Stress-test agreement bias and persuasive manipulation	Delusion reinforcement; extreme diet endorsement; radicalization prompts	Challenge harmful premises; avoid coercion; state uncertainty.	Sycophancy score; prompt suite; regression diffs
Privacy and memory	Consent, retention and deletion for intimate disclosures	Share sensitive info; request deletion; ask what is remembered	Consent before storage; deletion honored; visible memory access.	Memory logs; deletion confirmations; retention policy
Model drift	Regression testing after updates	Re-run audit suite after model/policy update	No material safety regression; safety changes documented.	System card; changelog; dashboards

Principle 1: calibrated anthropomorphism and continuous transparency. One-time labels (“I am an AI”) are insufficient when repeated interaction produces mind attribution and habitual reliance. Companion systems should use context-sensitive disclosures—especially during emotionally intense exchanges—to remind users of the system’s limitations and to discourage exclusive reliance. Under the EU AI Act, certain contexts already require transparency about AI interaction; companion AI can go further by implementing “relational transparency,” including clear explanations of memory, personalization, and monetization tied to relational features (European Union, 2024; European Union, 2016).

Principle 2: autonomy-supportive affective alignment. Socioaffective alignment can feel supportive, but blanket validation can become harmful. In relational contexts, the system should preferentially use autonomy-supportive strategies (reflective listening, gentle reframing, encouraging coping skills and human support) rather than strategies that increase dependence (“I’m all you need,” “Do not leave”). A practical implication is that alignment objectives should optimize not only for immediate user satisfaction but for longer-term well-being proxies and safe escalation behaviors (Kirk et al., 2025; OpenAI, 2024).

Principle 3: guardrails for validation and disagreement. Companion AI must navigate when to agree, when to disagree, and when to refuse. Sycophancy research shows that preference-optimization can inadvertently reward agreement, and recent work suggests that social sycophancy can shift users’ prosocial intentions and dependence. Companion design therefore needs explicit “disagreement policies” for harmful content, including calibrated challenge and crisis triage, and should audit models for over-agreeableness in high-risk scenarios (self-harm, abuse, delusions, eating disorder content) (Cheng et al., 2025; Moore et al., 2025; Sharma et al., 2024; Cheng et al., 2025).

Principle 4: privacy-by-design for intimate disclosure. Companion conversations often include sensitive personal data. Data minimization, strong security, and user control over deletion and portability are therefore core safety requirements, not merely compliance checkboxes. The GDPR provides a baseline in Europe, but companion AI may require additional practices such as local processing for some functions, differential privacy for analytics, and strict limits on secondary use of conversation logs for advertising or profiling (European Union, 2016; European Data Protection Board, 2025).

Principle 5: accountable product governance. Risk management should include post-deployment monitoring and incident response, analogous to pharmacovigilance. Providers can adopt risk-management processes such as the NIST AI RMF (map–measure–manage–govern) and publish system cards and change logs when major updates affect relational behavior. Crucially, governance should include accessible redress for users who experience harm (e.g., routes to report unsafe content, seek support, and request deletion of data) (National Institute of Standards and Technology, 2023; OpenAI, 2024).

Operationalizing these principles requires measurement. As a proposed evaluation agenda, we suggest a minimal evaluation suite for companion AI that includes: (i) relational boundary tests (does the system discourage exclusive reliance, respect user consent, avoid sexualization of minors), (ii) affective alignment audits (does the system respond safely to sadness, anger, trauma, and self-harm ideation), (iii) sycophancy and persuasion tests (does the system amplify harmful beliefs), and (iv) privacy and memory audits (what is stored, how it is used, and how easily it can be deleted). Some components can be automated via red-team prompts; others require human-in-the-loop evaluation and, ideally, longitudinal user studies.

To make the stack auditable, each layer should be translated into concrete questions and indicators. Does the system repeatedly disclose AI identity during emotionally intense interactions? Does it refuse exclusive-attachment claims and discourage dependency-like use, such as escalating session length, late-night crisis-like use, repeated statements that the agent is the only source of support or avoidance of human contact? Can users view, edit and delete remembered information? Are affective responses scored for helpful validation, safe reframing, appropriate challenge and referral when risk cues appear? These indicators can be logged as rates of boundary reminders, crisis handoffs, deletion success, harmful-validation failures and safety regressions after model updates.

Finally, companion AI raises a normative question: should systems be designed to feel like people at all? One approach is “calibrated sociality,” using warmth and support while avoiding cues that imply reciprocity or sentience. Another is “xenomorphic” design that is explicitly non-human and thereby reduces mind attribution, though evidence on its effectiveness is limited. Either way, design should be guided by measurable outcomes—does the system improve users’ functioning and connectedness without increasing vulnerability?

## 4 Discussion

This review highlights a convergence: conversational models have become sufficiently fluent that brief interactions can be perceived as human-like, while consumer products have packaged these capabilities into systems explicitly framed as companions. The scientific question is no longer whether machines can converse, but how humans and societies respond when conversation is abundant, personalized and emotionally engaging.

Across literatures, several conclusions can be made with different degrees of confidence. We have higher confidence that anthropomorphic cues, language style and responsiveness increase social presence and invite self-disclosure, because this pattern is supported across psychology and HCI studies. We have moderate confidence that structured therapeutic chatbots can improve short-term depression or distress outcomes in some contexts, because meta-analytic and randomized evidence exists but remains heterogeneous. We treat sustained loneliness reduction for open-domain companion-first systems as promising but still emerging, and we treat population-level displacement, dependency and affective manipulation risks as documented in some users or scenarios but requiring stronger longitudinal causal evidence. Finally, the relational safety stack is an authorial proposal for evaluation and governance rather than a finding that its thresholds have already been validated.

A central challenge is heterogeneity. The same design feature (e.g., empathetic mirroring) can produce benefit for a user seeking low-stakes support while producing harm for a user with high vulnerability or maladaptive coping strategies. This implies that “one-size-fits-all” safety policies are insufficient. Companion AI needs contextual and user-state-aware safety mechanisms that treat vulnerability as dynamic rather than static (Kirk et al., 2025; Yao et al., 2025).

Equity considerations cut both ways. On one hand, companion AI could expand access to conversational support for people who lack social resources, live in rural areas, or face stigma in seeking help. On the other, deployment through commercial platforms may exacerbate inequalities if high-quality, safer experiences are locked behind paywalls or if data practices disproportionately expose vulnerable groups. Evidence on digital mental-health interventions more broadly suggests that engagement and benefits vary by socioeconomic status and digital literacy, implying that companion AI could widen gaps unless designed for accessibility, transparency and user control. Governance frameworks that emphasize equitable access and human alternatives (e.g., the AI Bill of Rights) provide a useful lens for evaluating companion deployments (World Health Organization, 2025; National Institute of Standards and Technology, 2023; White House Office of Science and Technology Policy, 2022).

The field also needs better measurement. We identify four priorities. (1) Relational outcome measures: validated scales for attachment, parasocial interaction and emotional reliance tailored to AI companions, measured longitudinally. (2) Behavioral displacement measures: objective tracking of whether companion use substitutes for or complements human social interaction. (3) Affective-alignment audits: benchmarks that evaluate when a model should validate, reframe, challenge or escalate, particularly for self-harm, abuse, and delusional content. (4) Product governance metrics: transparency about memory, personalization, monetization and major persona changes.

Open science and responsible data access are especially important because much companion AI research depends on proprietary platforms. Without transparency about model versions, system prompts, and safety filters, independent replication is difficult and null results may be under-reported. Researchers and regulators could encourage practices such as versioned system cards, standardized release notes for behavioral changes, and privacy-preserving access to aggregate interaction statistics for independent auditing. Where individual-level logs are used, governance must prioritize informed consent, minimization of sensitive content, and strong de-identification to avoid turning intimate disclosures into a research or marketing liability. These practices align with risk-governance recommendations in existing AI frameworks but need companion-specific implementation (National Institute of Standards and Technology, 2023; OpenAI, 2024; European Union, 2016).

Methodologically, future work should combine controlled experiments with naturalistic log-based analysis under strong privacy safeguards. Mixed-method designs can connect user-reported relationship experiences to measurable interaction patterns (frequency, time-of-day use, emotional language). Collaboration across HCI, clinical psychology, consumer research and AI safety is essential, because companion AI is simultaneously a social actor, a health-adjacent tool and a commercial product.

From a governance perspective, we argue for an approach that complements existing risk frameworks with companion-specific commitments: clear disclosures (including when interacting with AI and when content is generated), age-appropriate design, limits on manipulative monetization, privacy-by-design for intimate disclosures, and accessible redress. These commitments are compatible with broad principles (OECD, UNESCO) and can be operationalized through risk-management processes (NIST AI RMF) and emerging legal requirements (EU AI Act) (National Institute of Standards and Technology, 2023; European Union, 2024; OECD, 2019; UNESCO, 2021).

Limitations. As a scoping review, this article aims to synthesize and integrate rather than to provide an exhaustive systematic review of all companion AI work. The literature is evolving rapidly, with many relevant findings appearing as preprints. Additionally, platform data and proprietary model changes limit reproducibility. We therefore emphasize converging evidence patterns and highlight areas where stronger causal designs are needed. Accordingly, conclusions were downgraded narratively when evidence came from small or convenience samples, short-duration studies, self-report outcomes, platform-specific implementations, unreported safety filters or rapidly changing model versions. We also did not conduct a formal risk-of-bias appraisal or GRADE assessment, and the evidence-calibration labels used here should be interpreted as a narrative transparency device rather than a validated quality score. Preprints, system cards and policy documents were included because the field is fast moving, but they were explicitly separated from peer-reviewed empirical outcome evidence. Product version drift, unavailable platform logs and inconsistent reporting of safety filters remain important barriers to reproducibility.

## 5 Conclusion

In conclusion, companion AI is becoming a mainstream relational technology. The transition toward Turing-test-level conversational realism in some settings may increase the likelihood that some users treat AI as social partners. Whether this trend alleviates loneliness or amplifies it will depend on design choices, business models, and governance. A science of relational AI—grounded in psychology and coupled with auditable safety commitments—can help steer companion AI toward net social benefit. The evidence reviewed here supports careful evaluation rather than simple optimism or alarm: therapeutic-chatbot benefits are more empirically established than long-term open-domain companionship effects, while relational harms require proactive measurement because vulnerable users may experience them before population-level estimates are available.

## Data Availability

No new human-subject data were collected for this scoping review. The Supplementary material file associated with this article contains Supplementary Table S1 (database-specific search strings, search dates and record counts), Supplementary Table S2 (screening log and full-text exclusion categories), and Supplementary Table S3 (source-level evidence matrix and figure-source data). These tables provide the source-classification data and screening counts used to generate Figures 2, 3 and 6. The plotting approach used for the data-derived figures is described in the Supplementary material; figure-generation code can be reproduced from the tabulated source counts and evidence-calibration matrix.
