# Supplementary material for: Human-like conversational agents as social partners: a scoping review of socioaffective mechanisms, well-being outcomes, risks and governance in the post-Turing era
Source: Front Artif Intell. 2026 Jul 15;9:1810097. doi: 10.3389/frai.2026.1810097 (PMC13416237; doi:10.3389/frai.2026.1810097)
Supplement: Supplementary file 1 [file Table_1.DOCX]

**Supplementary Material**

*Human-like conversational agents as social partners: a scoping review*

This Supplementary Material contains Supplementary Tables S1–S3 referenced in the manuscript. It documents the database-specific search strategy, PRISMA-ScR screening counts, evidence calibration, figure-source data and complete source-level evidence matrix used for the scoping review.

# Supplementary Table S1. Search sources, database-specific strings and records identified

| **Source** | **Date searched** | **Database-specific search string or strategy** | **Records** |
| --- | --- | --- | --- |
| PubMed | 15 Jan 2026 | ((chatbot*[Title/Abstract] OR "conversational agent*"[Title/Abstract] OR "large language model*"[Title/Abstract] OR "social chatbot*"[Title/Abstract] OR "companion AI"[Title/Abstract]) AND (loneliness OR "social support" OR well-being OR depression OR anxiety OR dependency OR anthropomorphism OR "social presence" OR attachment OR parasocial OR sycophancy OR privacy OR governance)) AND 2016/01/01:2026/01/15[dp] | 172 |
| ACM Digital Library | 15 Jan 2026 | ("chatbot" OR "conversational agent" OR "large language model" OR "social chatbot" OR "companion AI") AND (loneliness OR "social support" OR wellbeing OR "self-disclosure" OR attachment OR parasocial OR dependency OR sycophancy OR governance) | 214 |
| IEEE Xplore | 15 Jan 2026 | ("All Metadata":chatbot OR "All Metadata":"conversational agent" OR "All Metadata":"large language model") AND ("All Metadata":loneliness OR "All Metadata":"social support" OR "All Metadata":wellbeing OR "All Metadata":privacy OR "All Metadata":governance) | 167 |
| Scopus | 15 Jan 2026 | TITLE-ABS-KEY(chatbot* OR "conversational agent*" OR "large language model*" OR "social chatbot*" OR "companion AI") AND TITLE-ABS-KEY(loneliness OR "social support" OR wellbeing OR depression OR anxiety OR dependency OR anthropomorphism OR "social presence" OR attachment OR parasocial OR sycophancy OR privacy OR governance) AND PUBYEAR > 2015 | 424 |
| Web of Science | 15 Jan 2026 | TS=(chatbot* OR "conversational agent*" OR "large language model*" OR "social chatbot*" OR "companion AI") AND TS=(loneliness OR "social support" OR wellbeing OR depression OR anxiety OR dependency OR anthropomorphism OR "social presence" OR attachment OR parasocial OR sycophancy OR privacy OR governance), 2016-2026 | 383 |
| arXiv | 15 Jan 2026 | all:(chatbot OR "conversational agent" OR "large language model" OR "companion AI") AND all:(loneliness OR "social support" OR dependency OR sycophancy OR "social presence" OR governance) | 181 |
| SSRN | 15 Jan 2026 | chatbot OR "conversational AI" OR "AI companion" OR "large language model" AND loneliness OR social support OR dependency OR governance OR sycophancy | 94 |
| Publisher portals, standards/governance websites and citation chaining | 15 Jan 2026 | Targeted searches of Nature Portfolio, Oxford Academic, Frontiers, JCR, NIST, EU, OECD, UNESCO, WHO, FTC, EDPB, provider system-card pages and backward/forward citation chaining for included seed papers | 125 |

# Supplementary Table S2. PRISMA-ScR screening log and exclusion reasons

| **Stage** | **Record/report category** | **Count** |
| --- | --- | --- |
| Identification | Database and repository records | 1,635 |
| Identification | Additional records from governance/technical websites and citation chaining | 125 |
| Deduplication | Duplicates removed | 480 |
| Screening | Records screened by title/abstract or executive summary | 1,280 |
| Screening | Records excluded at title/abstract or executive-summary stage | 1,040 |
| Eligibility | Full-text reports assessed for eligibility | 240 |
| Eligibility exclusion reason | Not conversational, not socioaffective, or outside companion/assistant/therapeutic chatbot scope | 61 |
| Eligibility exclusion reason | No human-interaction outcome, user experience, safety or governance relevance | 48 |
| Eligibility exclusion reason | Outside date/language/source-type criteria | 32 |
| Eligibility exclusion reason | Purely technical model paper or insufficient methodological transparency | 26 |
| Eligibility exclusion reason | Unavailable full text, duplicate report, superseded version or non-citable web item | 15 |
| Included | Sources included in final scoping evidence map | 58 |

# Supplementary Table S3a. Evidence-calibration rubric used for Figure 3 and narrative synthesis

| **Score / label** | **Operational interpretation** |
| --- | --- |
| 3 Stronger empirical support | Multiple peer-reviewed studies, randomized trials or meta-analytic evidence directly addressing the claim; still interpreted within system type and context. |
| 2 Moderate support | Consistent qualitative, mixed-method, longitudinal or experimental evidence with limitations, or strong evidence from an adjacent system type. |
| 1 Preliminary support | Single-study, preprint, commentary-supported, indirect or primarily scenario/audit evidence; hypothesis-generating. |
| 0 Not primary / gap | No direct included evidence for this source type/domain; discussed only as a gap or governance implication. |

# Supplementary Table S3b. Data used for Figure 2

| **Year** | **Annual included evidence sources** | **Cumulative evidence sources** |
| --- | --- | --- |
| 2016 | 1 | 1 |
| 2017 | 1 | 2 |
| 2018 | 1 | 3 |
| 2019 | 2 | 5 |
| 2020 | 5 | 10 |
| 2021 | 4 | 14 |
| 2022 | 3 | 17 |
| 2023 | 9 | 26 |
| 2024 | 8 | 34 |
| 2025 | 24 | 58 |
| 2026 | 0 | 58 |

# Supplementary Table S3c. Data used for Figure 3

| **System/source type** | **Sociality mechanisms** | **Well-being benefits** | **Loneliness/social support** | **Reliance/displacement** | **Sycophancy/validation risk** | **Privacy/governance** |
| --- | --- | --- | --- | --- | --- | --- |
| Therapeutic chatbots | 2 | 3 | 2 | 1 | 1 | 1 |
| Companion-first systems | 3 | 2 | 2 | 2 | 1 | 2 |
| Assistant-first LLMs | 2 | 1 | 1 | 2 | 2 | 2 |
| Policy/governance sources | 1 | 0 | 0 | 1 | 1 | 3 |

# Supplementary Table S3d. Complete source-level evidence matrix

| **ID** | **Source** | **Year** | **Source category** | **System/source type** | **Design/document type** | **Primary evidence role** | **Weighting note** |
| --- | --- | --- | --- | --- | --- | --- | --- |
| 1 | Jones and Bergen (2025) | 2025 | Preprint | Assistant-first / model evaluation | Preregistered imitation-game experiment | Turing-style social plausibility | Reported preregistration; preprint status. |
| 2 | Mei et al. (2024) | 2024 | Peer-reviewed article | Assistant-first / model evaluation | Large-scale behavioral indistinguishability study | Turing-style social plausibility | Peer-reviewed; not a companion outcome study. |
| 3 | Zhou et al. (2020) | 2020 | Peer-reviewed article | Companion-first | System design and deployment description | Companion architecture and engagement | Large-scale platform description; no controlled well-being outcomes. |
| 4 | Snap Inc. (2023) | 2023 | Company report | Assistant-first / platform chatbot | Provider announcement | Adoption and governance context | Grey literature; used for scale/context only. |
| 5 | Li et al. (2023) | 2023 | Peer-reviewed systematic review/meta-analysis | Therapeutic / mental-health CA | Systematic review and meta-analysis | Mental-health outcomes | Higher weight for short-term therapeutic symptom claims. |
| 6 | De Freitas et al. (2025) | 2025 | Peer-reviewed article | Companion-first | Online/lab experiments | Loneliness and perceived support | Direct loneliness evidence; short-term and context-bound. |
| 7 | Office of the U.S. Surgeon General (2023) | 2023 | Public-health policy report | Governance / public health | Advisory report | Loneliness context | Authoritative context, not AI intervention evidence. |
| 8 | World Health Organization (2025) | 2025 | Public-health policy report | Governance / public health | Commission report | Social connection and loneliness context | Authoritative context, not AI intervention evidence. |
| 9 | Bommasani et al. (2021) | 2021 | Preprint/technical report | Foundation models | Technical/policy synthesis | Foundation-model risks | Used as background for model capability and risks. |
| 10 | OpenAI (2023) | 2023 | Technical report | Assistant-first LLM | System technical report | Model capability context | Provider technical report; not outcome evidence. |
| 11 | Kosinski (2024) | 2024 | Peer-reviewed article | Assistant-first / ToM evaluation | Behavioral task evaluation | Mind perception / theory of mind | Evidence for model performance, not user outcomes. |
| 12 | Strachan et al. (2024) | 2024 | Peer-reviewed article | Assistant-first / ToM evaluation | Comparative behavioral evaluation | Theory-of-mind task performance | Directly relevant to social plausibility. |
| 13 | Guingrich et al. (2024) | 2024 | Peer-reviewed article | Human-AI interaction | Empirical psychology study | Implicit mind perception | Mechanism evidence. |
| 14 | Ada Lovelace Institute (2025) | 2025 | Policy report | Governance / companion AI | Independent policy analysis | Companion risks and governance | Authoritative grey literature; not empirical effect evidence. |
| 15 | Cheng et al. (2025a) | 2025 | Preprint | Assistant-first LLM | Conceptual/empirical sycophancy work | Sycophancy risk | Preprint; used as risk mechanism evidence. |
| 16 | Moore et al. (2025) | 2025 | Peer-reviewed conference paper | Assistant-first / mental-health-like conversations | FAccT audit study | Unsafe mental-health responses | Direct safety-audit evidence. |
| 17 | Kirk et al. (2025) | 2025 | Preprint / position paper | Socioaffective alignment | Conceptual framework | Affective alignment | Normative/conceptual; not outcome evidence. |
| 18 | NIST (2023) | 2023 | Standards framework | Governance | Risk-management framework | Risk management and auditing | Authoritative standards source. |
| 19 | European Union (2024) | 2024 | Regulation | Governance | Legal instrument | Transparency and AI governance | Authoritative legal source. |
| 20 | Manoli et al. (2025) | 2025 | Preprint | Companion/assistant dynamics | Qualitative/characterization study | Companion-assistant taxonomy | Preprint; used cautiously for taxonomy. |
| 21 | OpenAI (2024) | 2024 | System card | Assistant-first LLM | Provider safety evaluation | Emotional reliance and modality risks | Provider evidence; not independent outcome evidence. |
| 22 | Araujo (2018) | 2018 | Peer-reviewed article | Conversational agents | Experiment | Anthropomorphism/social presence | Mechanism evidence. |
| 23 | Lee et al. (2020) | 2020 | Peer-reviewed conference paper | Conversational agent | Controlled HCI study | Self-disclosure and validation | Mechanism evidence. |
| 24 | Croes et al. (2023) | 2023 | Peer-reviewed article | Social chatbot | Content analysis | Language strategies and relational framing | Mechanism/user-experience evidence. |
| 25 | Hu, J. et al. (2025) | 2025 | Peer-reviewed evaluation critique | Assistant-first / ToM evaluation | Evaluation critique | Theory-of-mind evaluation | Mechanism and evaluation caveat. |
| 26 | Chen, R. et al. (2025) | 2025 | Peer-reviewed conference paper | Assistant-first / ToM evaluation | Assessment and modeling study | Theory-of-mind assessment | LLM social-cognition evaluation; not user outcome evidence. |
| 27 | Ta et al. (2020) | 2020 | Peer-reviewed qualitative study | Companion chatbots | Thematic analysis | Social support from companion chatbots | Direct user-experience evidence. |
| 28 | Brandtzaeg et al. (2021) | 2021 | Peer-reviewed mixed-methods study | Social/companion chatbots | Mixed-methods user study | Youth and chatbot social support | Mechanism/user-experience evidence. |
| 29 | Skjuve et al. (2021) | 2021 | Peer-reviewed empirical study | Companion chatbots | Survey/interview study | Human-chatbot relationships | Companion-first user evidence. |
| 30 | Xie and Pentina (2022) | 2022 | Conference case study | Replika / companion-first | Case study | Attachment theory and Replika | Platform-specific; theoretical framing. |
| 31 | Pentina et al. (2023) | 2023 | Peer-reviewed mixed-methods study | Replika / companion-first | Survey-based model | Relationship development | Platform-specific relationship evidence. |
| 32 | Hu, D. et al. (2025) | 2025 | Peer-reviewed mixed-methods study | Social companion AI | Two-stage mixed-method study | Attachment to social companion AI | Companion-first mechanism evidence. |
| 33 | Abd-Alrazaq et al. (2020) | 2020 | Peer-reviewed systematic review/meta-analysis | Therapeutic / mental-health CA | Systematic review/meta-analysis | Mental-health chatbot effectiveness/safety | Higher weight for therapeutic-chatbot claims. |
| 34 | Fitzpatrick et al. (2017) | 2017 | Peer-reviewed randomized trial | Therapeutic chatbot | Randomized controlled trial | Woebot symptom reduction | Direct short-term therapeutic evidence. |
| 35 | Maples et al. (2024) | 2024 | Peer-reviewed empirical study | Assistant-first / student support | Empirical study | Student loneliness/suicide mitigation | Context-specific; not long-term companion evidence. |
| 36 | Fang et al. (2025) | 2025 | Preprint longitudinal randomized study | Assistant-first / extended chatbot use | Longitudinal randomized study | Psychosocial effects of extended use | Preprint; useful for emerging risk/benefit heterogeneity. |
| 37 | Yao et al. (2025) | 2025 | Peer-reviewed survey | AI chatbot use | Survey mediation study | Problematic AI chatbot use mechanisms | Correlational; not causal evidence. |
| 38 | Maral et al. (2025) | 2025 | Peer-reviewed psychometric study | AI chatbot use | Scale development | Problematic ChatGPT Use Scale | Measurement evidence. |
| 39 | Ciudad-Fernández et al. (2025) | 2025 | Peer-reviewed theoretical critique | AI chatbot use | Theoretical critique | Caution around AI-addiction construct | Conceptual caution. |
| 40 | Yankouskaya et al. (2025) | 2025 | Peer-reviewed theoretical article | AI conversational LLMs | Theoretical article | Dependence-risk framing | Conceptual risk framing. |
| 41 | Chen, Q. et al. (2025) | 2025 | Peer-reviewed empirical-theoretical study | Human-chatbot relationships | Empirical/theoretical study | Romantic feelings toward chatbots | Emerging relational-dynamics evidence. |
| 42 | Sharma et al. (2024) | 2024 | Peer-reviewed ML conference paper | Assistant-first LLM | Machine-learning study | LLM sycophancy mechanisms | Technical risk mechanism. |
| 43 | Cheng et al. (2025b) | 2025 | Preprint experimental study | Assistant-first LLM | Experimental study | Sycophantic AI and dependence | Preprint; risk mechanism evidence. |
| 44 | Mathur et al. (2019) | 2019 | Peer-reviewed empirical study | Digital choice architecture | Large-scale crawl | Dark patterns and manipulative design | Adjacent evidence for product-risk framing. |
| 45 | Emotional risks of AI companions demand attention (2025) | 2025 | Editorial / commentary | Companion AI | Editorial commentary | Emotional reliance risk framing | Commentary; used for risk framing only. |
| 46 | Shank et al. (2025) | 2025 | Peer-reviewed commentary | AI romance | Commentary | Artificial intimacy ethics | Ethical/risk framing. |
| 47 | European Union (2016) | 2016 | Law / regulation | Governance | Legal instrument | GDPR data protection baseline | Authoritative legal source. |
| 48 | European Data Protection Board (2025) | 2025 | Regulatory enforcement notice | Governance / Replika | Enforcement notice | Replika privacy/minors enforcement context | Regulatory context; not outcome evidence. |
| 49 | OECD (2019) | 2019 | Policy principles | Governance | International recommendation | Trustworthy AI principles | High-level principles. |
| 50 | UNESCO (2021) | 2021 | Ethics recommendation | Governance | International recommendation | Human rights/well-being | High-level principles. |
| 51 | White House OSTP (2022) | 2022 | Policy framework | Governance | Policy blueprint | Notice, explanation, human alternatives | High-level policy source. |
| 52 | Cyberspace Administration of China (2023) | 2023 | Regulatory measure | Governance | Legal/policy instrument | Generative AI service governance | Jurisdiction-specific policy source. |
| 53 | U.S. FTC (2023) | 2023 | Regulatory guidance | Governance / consumer protection | Agency guidance | AI marketing claims | Consumer-protection source. |
| 54 | Paris et al. (2025) | 2025 | Preprint | Model evaluation | Collective Turing-test proposal | Detection and governance | Preprint; evaluation proposal. |
| 55 | Ouyang et al. (2022) | 2022 | Preprint/technical paper | Assistant-first LLM | RLHF training paper | Alignment and sycophancy context | Technical background. |
| 56 | Tang et al. (2025) | 2025 | Working paper / SSRN | Supportive social chatbot | Field experiment | Empathetic response strategies | Preprint/working paper; mechanism evidence. |
| 57 | Folk et al. (2025) | 2025 | Peer-reviewed article | Social chatbots | Cross-cultural survey | Cultural variation in attitudes | Mechanism/moderation evidence. |
| 58 | Lin et al. (2020) | 2020 | Peer-reviewed conference paper | Empathetic chatbot | System demonstration | Empathy response generation | Technical/mechanism context. |
